# Obesity is a risk factor for intrahepatic cholangiocarcinoma progression associated with alterations of metabolic activity and immune status

**DOI:** 10.1038/s41598-021-85186-6

**Published:** 2021-03-12

**Authors:** Kyohei Yugawa, Shinji Itoh, Norifumi Iseda, Takeshi Kurihara, Yoshiyuki Kitamura, Takeo Toshima, Noboru Harada, Kenichi Kohashi, Shingo Baba, Kousei Ishigami, Yoshinao Oda, Tomoharu Yoshizumi, Masaki Mori

**Affiliations:** 1 Department of Surgery and Science, Graduate School of Medical Sciences, Kyushu University, Fukuoka, 812-8582 Japan; 2 Department of Anatomic Pathology, Graduate School of Medical Sciences, Kyushu University, Fukuoka, 812-8582 Japan; 3 Department of Clinical Radiology, Graduate School of Medical Sciences, Kyushu University, Fukuoka, 812-8582 Japan

**Keywords:** Tumour immunology, Surgical oncology, Cancer microenvironment, Cancer imaging

## Abstract

Body mass index (BMI) is well known to be associated with poor prognosis in several cancers. The relationship between BMI and the long-term outcomes of patients with intrahepatic cholangiocarcinoma (ICC) is incompletely understood. This study investigated the relationships of BMI with clinicopathological characteristics and patient outcomes, focusing on metabolic activity and immune status. The relationship between BMI and the maximum standardized uptake value (SUVmax) on fluorine-18 fluorodeoxyglucose (^18^F-FDG) positron emission tomography/computed tomography (PET/CT) was analyzed. In addition, immunohistochemistry was performed for programmed cell death-ligand 1 (PD-L1), cluster of differentiation 8 (CD8), and forkhead box protein P3 (Foxp3). Seventy-four patients with ICC were classified into normal weight (BMI < 25.0 kg/m^2^, n = 48) and obesity groups (BMI ≥ 25.0 kg/m^2^, n = 26), respectively. Serum carbohydrate antigen 19–9 levels were higher in the obesity group than in the normal weight group. Tumor size and the intrahepatic metastasis rate were significantly larger in the obesity group. Patients in the obesity group had significantly worse prognoses than those in the normal weight group. Moreover, BMI displayed a positive correlation with SUVmax on ^18^F-FDG PET/CT (n = 46, *r* = 0.5152). Patients with high ^18^F-FDG uptake had a significantly higher rate of PD-L1 expression, lower CD8 + tumor-infiltrating lymphocyte (TIL) counts, and higher Foxp3 + TIL counts. The elevated BMI might predict the outcomes of patients with ICC. Obesity might be associated with ICC progression, possibly through alterations in metabolic activity and the immune status.

## Introduction

Intrahepatic cholangiocarcinoma (ICC) is the second most common primary liver tumor after hepatocellular carcinoma (HCC) and a major cause of cancer mortality and morbidity worldwide^1^. ICCs are estimated to account for approximately 5%–15% of all primary liver cancers, and their incidence has been increasing worldwide^2^. Even after curative hepatic resection, patients experience high rates of recurrence and early local or distant metastases^3,4^. Therefore, predictive risk factors that influence recurrence among patients with ICC are urgently required.

Obesity is a major concern that is correlated with increasing risks of several comorbid disorders, including cancer^5,6^. Body mass index (BMI), which is calculated from weight and height, is highly correlated with body fat mass. BMI is also known to be associated with the prognosis of several cancers^7^. Obesity leads to metabolic disorders, which can cause chronic inflammation in association with immune cells^8,9^. We hypothesize that the metabolic disorders caused by obesity are associated with tumor progression via alterations of the immune status.

Positron emission tomography/computed tomography (PET/CT) using fluorine-18 fluorodeoxyglucose (^18^F-FDG) for metabolic assessment is valuable for cancer detection and the staging of various malignancies, including cholangiocarcinoma^10^. The classification of FDG uptake using PET/CT has been established to be useful in predicting prognosis. The maximum standardized uptake value (SUVmax) on ^18^F-FDG PET/CT is significantly correlated with microvascular invasion, poor differentiation, and hypoxia-inducible factor 1A (HIF-1A) and glucose transporter 1 (GLUT1) expression in HCC^11–13^.

Immune checkpoint inhibitors (ICIs) have been highlighted as effective treatments for several cancers. In tumor immune microenvironment, programmed cell death-ligand 1 (PD-L1) plays a crucial role in the tumor immunobiology of ICC^14,15^. Moreover, tumor-infiltrating lymphocytes (TILs) also play an important role in tumor progression. Recently, we and other studies reported that cluster of differentiation 8 (CD8) + and forkhead box protein P3 (Foxp3) + T lymphocytes are associated with a poor prognosis in ICC^15–17^.

^18^F-FDG PET/CT is significantly associated with the immune status in several cancers^18–20^. To the best of our knowledge, no previous study elucidated the relationships of BMI with SUVmax on ^18^F-FDG PET/CT and the immune status in patients with ICC. This study investigated the relationships of BMI with clinicopathological characteristics and patient outcomes, focusing on metabolic activity and the immune status.

## Results

In total, 74 patients were included in this study. Of the total patients, 51 men and 23 women were included. The median patient age was 66 years (range, 39–87 years). The median OS and RFS were 7.4 and 1.7 years, respectively. Seven patients had hepatitis B virus infection, and 6 patients had hepatitis C virus infection. Non-alcoholic fatty liver disease (NAFLD) activity score (NAS) was calculated using standard guidelines for histological scoring system^21^. From histological features and criteria of regular alcohol intake (less than 210 g/week for men and 140 g/week for women)^22^, 9 patients were diagnosed as non-alcoholic steatohepatitis (NASH). Ten patients were diagnosed with liver cirrhosis according to the pathological features.

Fifty-nine patients were treated via complete resection (R0), whereas 15 patients underwent nearly complete resection (R1). In our institution, lymph node dissection was performed when lymph node metastasis was suspected on preoperative abdominal CT^4^. Thirteen patients had lymph node metastasis according to pathological examinations.

In Japan, the accepted cut-off for the diagnosis of obesity is different from that set for Western countries, and no overweight class is defined^23^. Therefore, patients were categorized as normal weight and obesity groups according to the Japan Society for the Study of Obesity as normal weight (BMI < 25 kg/m^2^, n = 48) and with obesity (BMI ≥ 25 kg/m^2^, n = 26)^24^. Regarding ^18^F-FDG PET/CT analysis, data for 46 patients whose PET/CT images remained in the electronic medical records were analyzed to examine the relationships of BMI with SUVmax and TIL counts.

### BMI and clinicopathological factors

The median BMI was 22.8 kg/m^2^ (range, 15.9–37.8 kg/m^2^), and the normal weight (BMI < 25.0 kg/m^2^) and obesity groups (BMI ≥ 25.0 kg/m^2^) included 48 (64.9%) and 26 patients (35.1%), respectively. The clinicopathological characteristics of patients in the normal weight and obesity groups are presented in Table 1. Regarding the factors related to metabolic syndrome, the rate of hypertension was more frequent in the obesity group than in normal weight group (29.2% vs. 73.1%, *P* = 0.0002). However, the presence of type 2 diabetes, fasting glucose and triglycerides levels were not significantly different between the two groups. Moreover, the presence of NASH was not significantly different between the two groups. Regarding oncological characteristics, serum carbohydrate antigen 19–9 (CA19-9) levels were higher in the obesity group than in the normal weight group (median, 21.5 U/mL vs. 42.9 U/mL, *P* = 0.0337). The obesity group had a significantly larger tumor size (median, 3.5 cm vs. 5.4 cm, *P* = 0.0292), and intrahepatic metastasis was more frequently observed in this group (20.8% vs. 57.7%, *P* = 0.0014).

**Table 1:** Comparison of clinicopathological factors between patients with normal weight and overweight following the hepatic resection of intrahepatic cholangiocarcinoma.

Factors	BMI < 25 kg/m^2^ (n = 48)	BMI ≥ 25 kg/m^2^ (n = 26)	*P* value
Age (yr)	66 (39–87)	67 (44–85)	0.5826
Sex (male/female)	33/15	18/8	0.9660
HBV (+ , %)	3 (6.3%)	4 (15.4%)	0.1999
HCV (+ , %)	6 (12.5%)	0 (0.0%)	0.6000
NASH (+ , %)	5 (10.4%)	4 (15.4)	0.5325
Hypertension (+ , %)	14 (29.2%)	19 (73.1%)	0.0002*
Type 2 diabetes (+ , %)	7 (14.6%)	7 (26.9%)	0.1957
Fasting glucose (mg/dL)	99 (71–247)	99.5 (89–277)	0.3779
Fasting triglycerides (mg/dL)	83 (33–384)	99.5 (60–290)	0.1453
Albumin (g/dL)	4.1 (3.3–5.3)	4.1 (3.5–4.8)	0.8290
Total bilirubin (mg/dL)	0.8 (0.2–1.7)	0.7 (0.3–1.1)	0.7025
Platelets (× 10^4^/μL)	19.8 (5.2–44.0)	17.7 (7.4–40.2)	0.7002
Total lymphocytes (× 10^3^/uL)	1.5 (0.4–4.0)	1.6 (0.8–2.8)	0.2458
CA19-9 (U/mL)	21.5 (0.6–40,795)	42.9 (0.6–21,100)	0.0337*
Tumor size (cm)	3.5 (0.5–12.0)	5.4 (1.8–10.0)	0.0292*
Poor differentiation (%)	30 (62.5%)	14 (53.9%)	0.4692
Microvascular invasion (%)	19 (39.6%)	16 (61.5%)	0.0709
Bile duct invasion (%)	18 (37.5%)	13 (50.0%)	0.2981
Intrahepatic metastasis (%)	10 (20.8%)	15 (57.7%)	0.0014*
Lymph node metastasis (%)	6 (12.5%)	7 (26.9%)	0.1196
Histological liver cirrhosis (%)	7 (14.6%)	3 (11.5%)	0.7145

Data are presented as n (%) or the median (range).

BMI, body mass index; CA19-9, carbohydrate antigen 19-9; HBV, hepatitis B virus; HCV, hepatitis C virus; NASH, non-alcoholic steatohepatitis, *P < 0.05.

### BMI and patient survival

**Figure 1:**
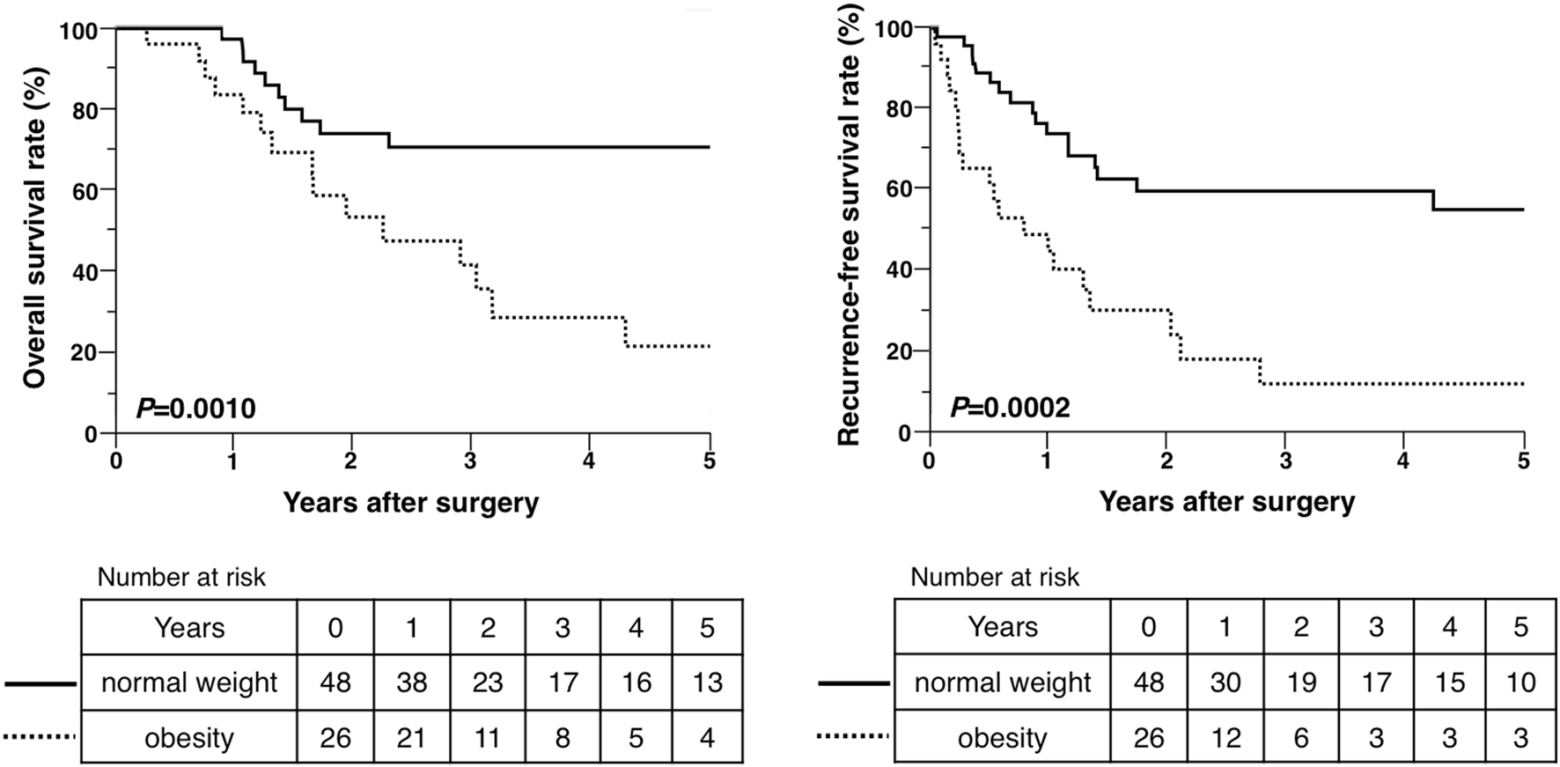
Overall and recurrence-free survival rates after hepatic resection for intrahepatic cholangiocarcinoma in patients in the normal weight and obesity groups.

The survival analysis was performed using the Kaplan–Meier method, which revealed that OS (log-rank *P* = 0.0010) and RFS (log-rank *P* = 0.0002) were significantly shorter in the obesity group than in the normal weight group. Median OS and RFS in the normal weight group were 9.6 [95% confidence interval (CI) = 8.2 to not reached] and 9.1 years (95% CI = 1.4 to not reached), respectively. Median OS and RFS in the obesity group were 2.3 (95% CI = 1.3–4.3) and 0.8 years (95% CI = 0.2–1.3), respectively (Fig. 1).

### Univariate and multivariate analyses of prognostic factors for OS and RFS

To evaluate clinical continuous variables, the following cutoffs were selected using receiver operating characteristic (ROC) curves: 69 years for patient age, 4.1 g/dL for serum albumin levels, 69.9 U/mL for serum CA19-9 levels, and 6.0 cm for tumor size. Univariate analysis identified high serum CA19-9 levels (≥ 69.9 U/mL), large tumor size (≥ 6.0 cm), positivity for intrahepatic and lymph node metastasis, and BMI ≥ 25.0 kg/m^2^ as significant prognostic factors for OS. Univariate analysis indicated that the significant prognostic factors for RFS were high serum CA19-9 levels, large tumor size (≥ 6.0 cm), positivity for microvascular invasion, intrahepatic node metastasis, and lymph node metastasis, and BMI ≥ 25.0 kg/m^2^. Multivariate analysis identified positivity for lymph node metastasis and BMI ≥ 25.0 kg/m^2^ as significant prognostic factors for OS and high serum CA19-9 levels positivity for microvascular invasion and lymph node metastasis, and BMI ≥ 25.0 kg/m^2^ as significant prognostic factors for RFS (Table 2).

**Table 2:** Univariate and multivariate analyses of risk factors for overall and recurrence-free survival following hepatic resection of intrahepatic cholangiocarcinoma.

Factors	Overall survival				Recurrence-free survival			
	Univariate analysis		Multivariate analysis		Univariate analysis		Multivariate analysis	
	HR (95% CI)	*P* value	HR (95% CI)	*P* value	HR (95% CI)	*P* value	HR (95% CI)	*P* value
Age (≥ 69)	2.05 (0.96–4.37)	0.0634			1.37 (0.71–2.63)	0.3417		
Male	1.07 (0.49–2.36)	0.8652			1.12 (0.57–2.22)	0.7346		
Albumin (< 4.1 g/dL)	2.03 (0.98–4.22)	0.0578			1.32 (0.70–2.50)	0.3926		
CA19–9 (≥ 69.9 U/mL)	2.62 (1.18–5.79)	0.0174*	2.26 (0.97–5.31)	0.0602	2.32 (1.20–4.49)	0.0127*	2.15 (1.07–4.32)	0.0317*
Tumor size (≥ 6.0 cm)	2.69 (1.26–5.76)	0.0109*	1.53 (0.62–3.76)	0.3568	2.62 (1.34–5.09)	0.0046*	1.70 (0.82–3.49)	0.1509
Poor differentiation	1.09 (0.52–2.27)	0.8196			1.38 (0.72–2.65)	0.3286		
Microvascular invasion ( +)	2.18 (0.97–4.86)	0.0583			2.65 (1.35–5.23)	0.0048*	2.57 (1.20–5.49)	0.0150*
Bile duct invasion ( +)	2.04 (0.94–4.39)	0.0698			1.29 (0.69–2.43)	0.4229		
Intrahepatic metastasis ( +)	2.28 (1.09–4.78)	0.0289*	1.02 (0.42–2.46)	0.9652	2.60 (1.36–4.96)	0.0037*	1.06 (0.50–2.24)	0.8833
Lymph node metastasis ( +)	3.39 (1.56–7.35)	0.0020*	3.17 (1.42–7.09)	0.0050*	2.40 (1.19–4.87)	0.0151*	2.10 (1.00–4.41)	0.0496*
Histological liver cirrhosis ( +)	1.38 (0.48–4.01)	0.5488			1.17 (0.46–3.01)	0.7433		
Insulin or insulin secretagogues ( +)	1.09 (0.46–2.55)	0.8500			0.85 (0.39–1.85)	0.6773		
NASH ( +)	0.97 (0.37–2.56)	0.9543			0.67 (0.24–1.93)	0.4627		
BMI ≥ 25.0 kg/m^2^	3.30 (1.56–6.98)	0.0018*	2.61 (1.12–6.07)	0.0261*	3.18 (1.68–6.00)	0.0004**	2.29 (1.15–4.54)	0.0182*

BMI, body mass index; CA19-9, carbohydrate antigen 19-9; CI, confident interval; HR, hazard ratio; NASH, non-alcoholic steatohepatitis, *P < 0.05 and **P < 0.001.

### ^18^F-FDG PET/CT analysis and the relationship between BMI and SUVmax

**Figure 2:**
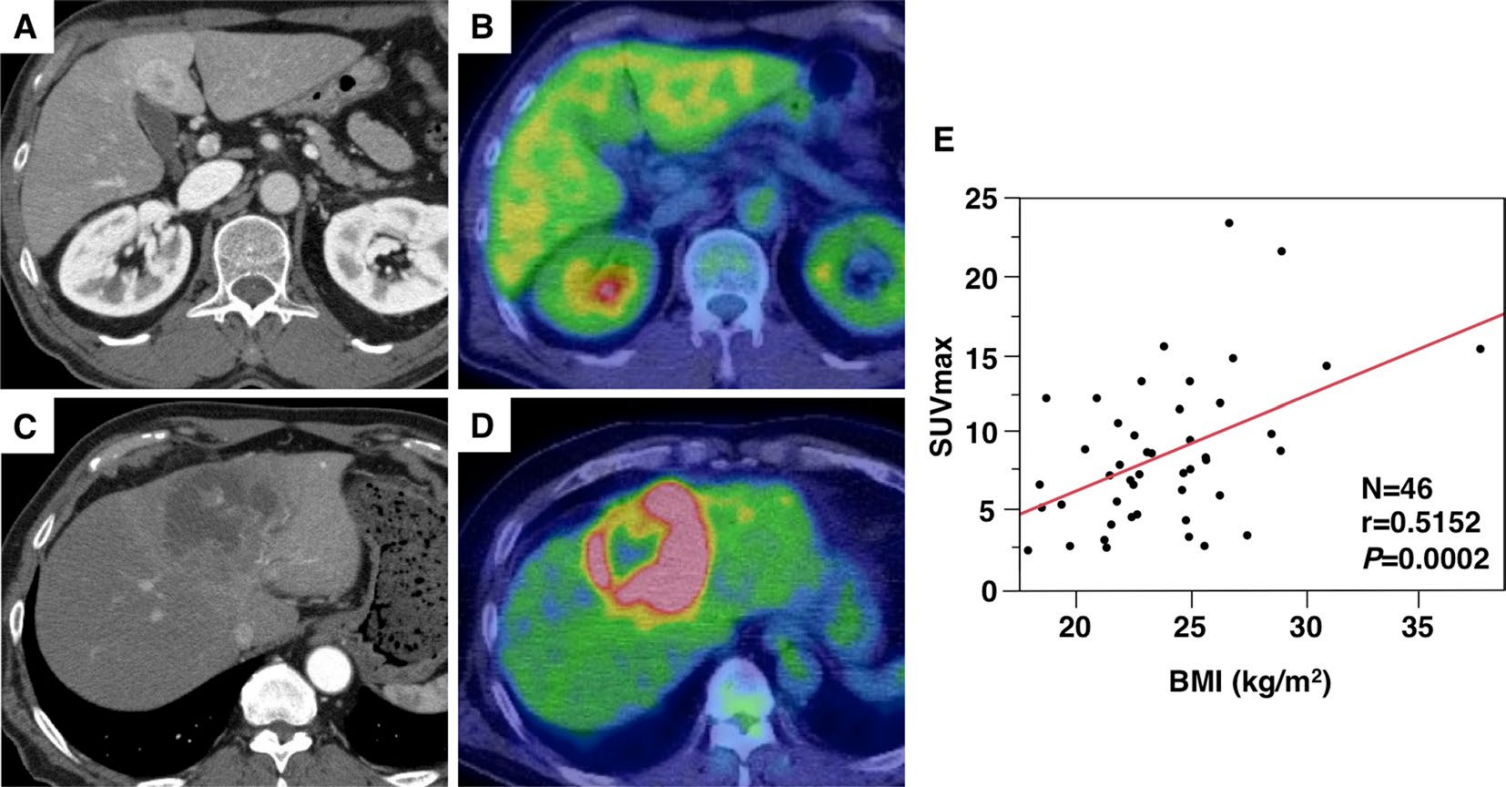
Representative positron emission tomography/computed tomography (PET/CT) images and the relationship between body mass index (BMI) and the maximum standardized uptake value (SUVmax) (n = 46). A patient with low SUVmax (< 6.79) on preoperative enhanced CT (**A**) and fluorine-18 fluorodeoxyglucose (^18^F-FDG) PET/CT (**B**). A patient with high SUVmax (≥ 6.79) on enhanced CT (**C**) and ^18^F-FDG PET/CT (**D**). The relationship between BMI and SUVmax (**E**).

The representative features of low and high SUVmax on ^18^F-FDG PET/CT are presented in Fig. 2A–D. The median SUVmax was 7.65 (range, 2.22–23.5), and the cutoff of SUVmax for postoperative prognosis was determined using ROC curve analysis (cutoff = 6.79, area under the ROC curve = 0.728, sensitivity = 87.5%, specificity = 53.3%, *P* = 0.0055, Supplementary Fig. 1). Among the 46 patients included in the analysis, 18 (39.1) and 28 patients (60.9%) comprised the low and high SUVmax groups, respectively. Kaplan–Meier curves for OS and RFS in patients with low and high SUVmax are presented in Supplementary Fig. 2. Both OS and RFS were worse in the high SUVmax group (log-rank *P* = 0.0208 and *P* = 0.0155, respectively). Next, the relationship between BMI and SUVmax in patients with ICC was clarified. Importantly, a statistically significant relationship was observed between BMI and SUVmax (*r* = 0.5152, *P* = 0.0002, Fig. 2E).

### Relationships among SUVmax, PD-L1 expression, and CD8 + and Foxp3 + TIL counts

**Figure 3:**
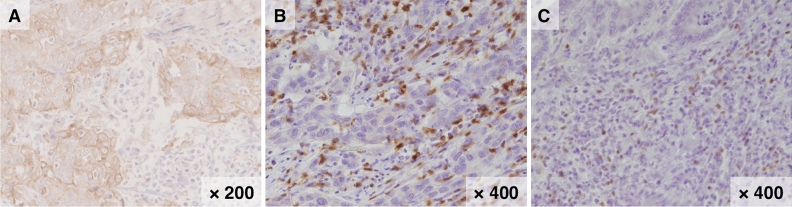
Representative features of programmed death-ligand 1 (PD-L1), cluster of differentiation 8 (CD8), and forkhead box protein P3 (Foxp3) immunohistochemical staining. Representative features of positive PD-L1 (× 200, **A**), CD8 (× 400, **B**), and Foxp3 + staining (× 400, **C**).

**Figure 4:**
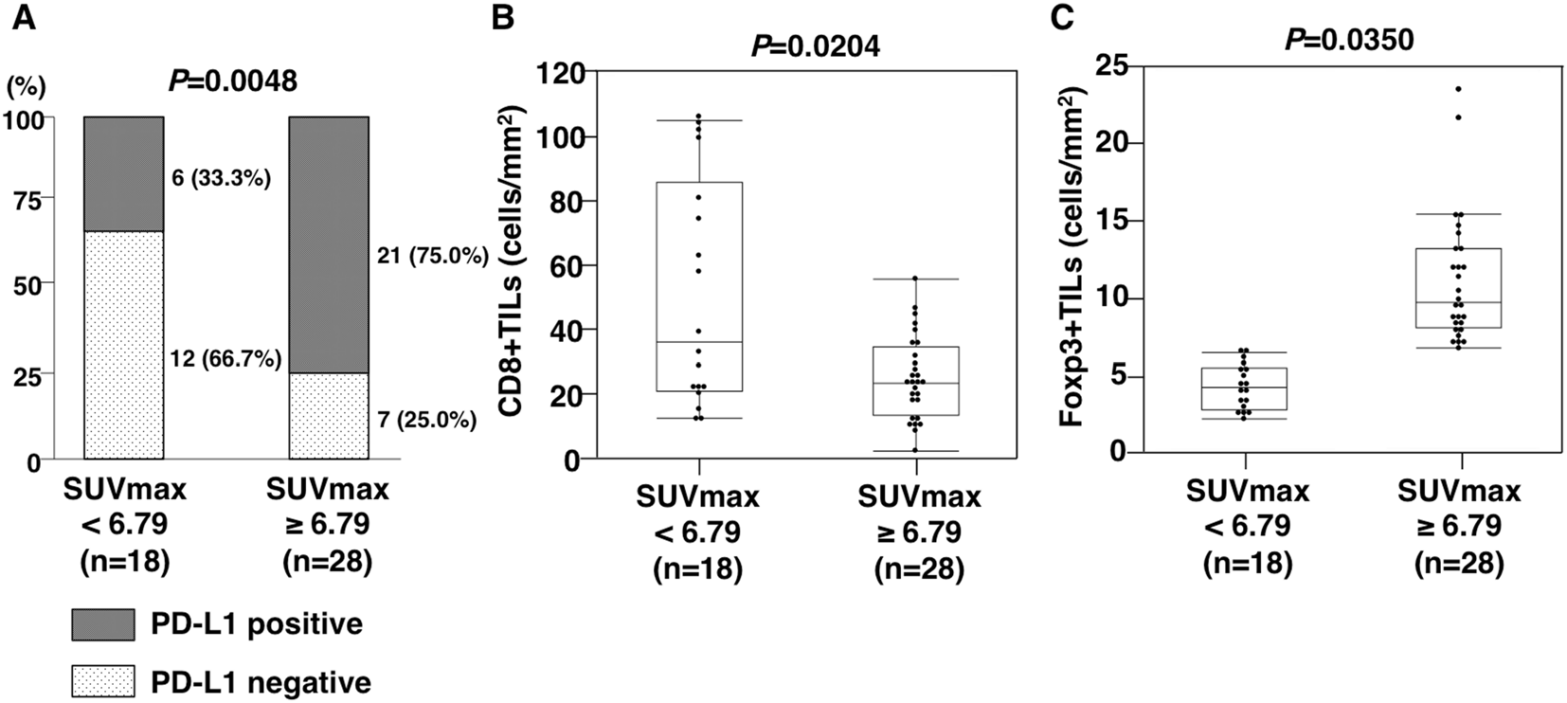
Relationships of fluorine-18 fluorodeoxyglucose (^18^F-FDG) uptake on positron emission tomography/computed tomography (PET/CT) with programmed death-ligand 1 (PD-L1) expression and cluster of differentiation 8 (CD8) + and forkhead box protein P3 (Foxp3) + tumor-infiltrating lymphocyte (TIL) counts. The rate of PD-L1 positivity in patients with a high maximum standardized uptake value (SUVmax) of ^18^F-FDG was significantly higher than those with low SUVmax (**A**). CD8 + TIL counts in patients with high SUVmax of ^18^F-FDG were lower than those with low SUVmax (**B**). Foxp3 + TIL counts in patients with high SUVmax of ^18^F-FDG were higher than those with low SUVmax (**C**).

To confirm whether metabolic activity was associated with the intratumoral immune status, the relationships of SUVmax with PD-L1 expression and CD8 + and Foxp3 + TIL counts in ICC tissues were clarified. Accordingly, immunohistochemical staining was performed to confirm PD-L1 expression and the detailed distribution of T lymphocytes in ICC. The representative features of immunohistochemical staining of PD-L1, CD8, and Foxp3 are presented in Fig. 3A–C. In this study, patients with PD-L1 + tumors had worse OS than those with PD-L1 − tumors (log-rank *P* = 0.0421, Supplementary Fig. 3). A statistically significant relationship was observed between SUVmax and PD-L1 expression (PD-L1 positivity: 33.3% vs. 75.0%, *P* = 0.0048, Fig. 4A). More importantly, patients with high SUVmax had significantly lower CD8 + and significantly higher Foxp3 + TIL counts than those with low SUVmax (median CD8 + TIL counts, 35.9 cells/mm^2^ vs. 23.1 cells/mm^2^, *P* = 0.0204; median Foxp3 + TIL counts, 3.1 vs. 5.9 cells/mm^2^, *P* = 0.0350, Fig. 4B,C).

## Discussion

In the current study, we found that increased BMI among patients undergoing curative resection for ICC was significantly associated with poor prognosis and an increased risk of recurrence. In addition, BMI significantly reflected high ^18^F-FDG attenuation on PET/CT in patients with ICC. Furthermore, high SUVmax was strongly correlated with positive PD-L1 expression on cancer cells, low CD8 + TIL counts, and high Foxp3 + TIL counts. Taken together, obesity is significantly associated with metabolic activity in ICC tissues, which might result in an altered immune status.

Our group and other researchers previously reported that obesity is associated with poor prognosis in HCC^25–27^. Thus, elevated BMI has been considered a predictive risk factor in several cancers, including HCC. More recently, Merath and corroborators demonstrated that increased BMI is independently associated with incremental increases in the risk of recurrence after curative-intent resection among patients with ICC^28^. Consistent with these results, we confirmed that high BMI was highly associated with poor prognosis among patients with ICC. In addition to our results, this finding suggests that metabolic disorders caused by obesity might be involved in the progression of ICC. However, in these previous studies, the detailed mechanisms by which BMI is associated with cancer progression were not elucidated. To our knowledge, this study is the first to demonstrate relationships between high BMI and ICC progression, focusing on ^18^F-FDG PET/CT as an indicator of the metabolic and immune status.

It is well known that obesity is strongly associated with NASH and type 2 diabetes^29^, and both were reported to be risk factors of ICC development^30,31^. However, in this population, NASH and diabetes were not significantly correlated with obesity, and were not prognostic factors for OS and RFS. Also the use of insulin or insulin secretagogues did not affect survival. Therefore, we hypothesized that obesity might lead to ICC progression via other mechanism(s) implicated in tumor metabolism and immune status.

Our results reinforce the hypothesis that some obesity-associated factors beyond inherent tumor characteristics influence the prognosis of patients with ICC. It is well recognized that DNA methylation, known as a critical regulatory mechanism of gene expression, is altered in some metabolically important genes among patients with high BMI^32,33^. Furthermore, adipocytes are associated with systemic hypoxia and cancer progression in the tumor microenvironment (TME)^34^. Several studies demonstrated that HIF-1A/GLUT1 signaling is related to ^18^F-FDG uptake among patients with cancer and metabolic disorder^18,35^. Moreover, glucose metabolism in tumor cells has been reported to play crucial roles in tumor progression by regulating local tumor immunity^36^. In this study, we evaluated the relationship between BMI and tumor metabolic activity, revealing a strong correlation between BMI and FDG uptake. These findings support that obesity-related metabolic, epigenetic, or hypoxic changes might induce the accumulation of FDG in the TME, resulting in an altered immune status in ICC.

More recently, ICIs have been highlighted as effective treatments for several cancers^37^. In HCC, our group and others previously reported that PD-L1 expression on cancer cells was associated with poor clinical outcomes^38–40^. We also showed that PD-L1 expression was a significant factor for overall survival in patients with ICC^15^. Moreover, high FDG uptake is significantly associated with high PD-L1 expression in esophageal squamous cell carcinoma and non-small cell lung cancer^18,41^. Consistent with these results, we observed a strong correlation between FDG uptake and PD-L1 expression, suggesting that ^18^F-FDG PET/CT is a great prognostic option for predicting sensitivity to ICIs.

Several studies reported that ^18^F-FDG PET/CT highly reflects the immune status defined by CD8 + and Foxp3 + TIL counts in several types of cancers. Our results indicated that the accumulation of FDG on PET/CT was positively correlated with Foxp3 regulatory TIL counts, but negatively correlated with CD8 cytotoxic TIL counts. Conversely, no relationships of PD-L1 expression on ICC cells with CD8 + and Foxp3 + TIL counts were identified in this study. These results suggest that PD-L1 expression on cancer cells might be regulated via metabolic mechanisms that are unique to ICC cells opposed to the effects of immune cells in the TME. Obesity-associated metabolic activity might result in PD-L1 expression, the suppression of CD8 + TIL counts, and the recruitment of Foxp3 + TILs, and these processes are considered to be involved in ICC progression.

This study had some potential limitations. First, this was a single-center and long-term retrospective study designed to examine prognostic factors influencing OS and RFS. This case series included patients who received postoperative adjuvant chemotherapy, which might have influenced their long-term outcomes. Second, our findings suggest that obesity can induce the production of certain mediators and regulate the anti-tumor immune response in ICC. However, we could not estimate a direct association of obesity with the immune status. Finally, other distributions of tumor-infiltrating immune cells and factors that affect PD-L1 expression were not clarified, and further work is required for elucidation.

In conclusion, we demonstrated that high BMI was an independent predictor of poor prognosis and risk of recurrence among patients with ICC after curative hepatic resection. Importantly, BMI was highly correlated with the accumulation of ^18^F-FDG on PET/CT. Moreover, high ^18^F-FDG uptake was positively correlated with PD-L1 expression and Foxp3 + TIL counts but negatively correlated with CD8 + TIL counts. These observations suggest that obesity is a risk factor for cancer progression in association with alterations of metabolic activity and immune status in ICC.

## Methods

### Patients and specimen preparation

All consecutive patients with ICC who had undergone hepatic resection from May 2000 to November 2019 at Kyushu University Hospital in Japan were enrolled. Patients who had undergone resection for primary ICC without preoperative chemotherapy or radiation were selected retrospectively. Preoperative and postoperative de-identified clinical information was obtained from electronic and paper records. The detailed surgical procedure and patient selection criteria for hepatic resection have been previously described^4,15^. Complete charts for clinical data were available for all patients, and informed consent was obtained from all patients to use their resected tissue for research.

To evaluate the histological features, the specimens were fixed in 10% formalin solution, embedded in paraffin, and sectioned into 4-µm-thick slices. One section from each patient was counterstained with hematoxylin and eosin for histological diagnoses^15^. In addition, the experimental protocol was approved by the ethics committee of Kyushu University. All methods were carried out in accordance with relevant guidelines and regulations of the Japanese government (Approval number: 2020-68 and 2020-180).

### ^18^F-FDG PET/CT

^18^F-FDG PET/CT was performed as previously described^42^. For each patient, the examination was performed after fasting for at least 4 h. ^18^F-FDG (185 MBq) was injected intravenously. Scans were conducted from the middle of the thigh to the top of the skull 60 min after FDG administration. FDG-PET/CT images were obtained using an integrated PET/CT scanner (Discovery STE; GE Medical Systems, Milwaukee, WI, USA) or Biograph mCT (Siemens Medical Solution, Erlangen, Germany). All emission scans were performed in the three-dimensional mode, and the acquisition time per bed position was 3 min for Discovery STE and 2 min for Biograph mCT. PET images were reconstructed using the ordered-subset expectation–maximization method (VUE Point Plus) with two full iterations of 28 subsets for Discovery STE, and the iterative True-X algorithm incorporates an additional specific correlation for the point-spread function. The full-width at half-maximum values of Discovery STE and Biograph mCT were 5.2 and 4.4 mm, respectively. Low-dose 16-slice CT (tube voltage, 120 kV; effective tube current, 30–250 mA; Discovery STE) and low-dose 32-slice CT (tube voltage, 120 kV; use of angular and longitudinal dose modulation; CAREDose4D; Biograph mCT) from the vertex to the proximal thigh were performed for the attenuation correction of PET images. CT scans were reconstructed via filtered black projection into 512 × 512-pixel images with a slice thickness of 5 mm to match the PET scan. FDG uptake in lesions was calculated using SUVmax, which was calculated using a dedicated workstation for each scanner.

### Immunohistochemical analysis

Immunohistochemical staining was carried out as previously described^15,43^. The sliced sections were deparaffinized in xylene and rehydrated in a graded ethanol series. Subsequently, the specimens were subjected to antigen retrieval [98 °C incubation via microwave for 20 min, Tris–EDTA buffer (pH 9.0) for CD8 and Foxp3 staining; 120 °C incubation via autoclave for 10 min, Tris–EDTA buffer (pH 9.0) for PD-L1 staining]. Next, the specimens were treated with 0.3% H_2_O_2_ for 5 min to inhibit endogenous peroxidase activity. The primary antibodies, including mouse monoclonal anti-CD8 (Clone C8/144B; Agilent Technologies, Santa Clara, CA, USA), mouse monoclonal anti-Foxp3 (236A/E7; Abcam, Cambridge, UK), and rabbit monoclonal anti-PD-L1 antibodies (E1L3N; Cell Signaling Technology, Danvers, MA, USA), were applied to the specimens at a dilution of 1:100 and incubated overnight at 4 °C. The next day, the specimens were incubated with labeled streptavidin–biotin for 1 h at room temperature. Color development was performed using 3,3′-diaminobenzidine, followed by counterstaining with Mayer’s hematoxylin.

The cutoff was set at 1% of total cancer cells for PD-L1 positivity according to our previous report^40^. The average CD8 + and Foxp3 + TIL counts were calculated for the five areas with the highest density of staining in the intratumoral area by counting CD8 + and Foxp3 + T cells under light microscopy (× 400 magnification). Immunohistochemical evaluations were performed independently by two observers (K.Y. and K.K.) who were blinded to the clinical background of the subjected patients.

### Statistical analysis

Statistical analysis was performed as previously reported^15^. Standard statistical analyses were used to evaluate descriptive statistics, including means, medians, frequencies, and percentages. Continuous variables were compared using the Mann–Whitney *U*-test and Kruskal–Wallis test. Categorical variables were compared using the χ^2^ test or Fisher’s exact test. Univariate and multivariate survival analyses were performed using Cox proportional hazard models. Cumulative overall survival (OS) and recurrence-free survival (RFS) rates were calculated using the Kaplan–Meier method, and differences between curves were evaluated using the log-rank test. OS was calculated as the time from the date of surgery to that of the last follow-up or death. To identify postoperative prognostic factors, several variables found to be independent in univariate analyses were included in the overall multivariate Cox proportional model to analyze both OS and RFS. All statistical tests were two-sided, and *P* < 0.05 indicated statistical significance. All statistical analyses were performed using JMP14 software (SAS Institute, Cary, NC, USA).

## Supplementary Information

Supplementary Information 1.

## Abbreviations

BMI: Body mass index
CA19-9: Carbohydrate antigen 19-9
CD8: Cluster of differentiation 8
CEA: Carcinoembryonic antigen
CI: Confidence interval
CT: Computed tomography
^18^F-FDG: Fluorine-18 fluorodeoxyglucose
Foxp3: Forkhead box protein P3
GLUT1: Glucose transporter 1
HCC: Hepatocellular carcinoma
HIF-1A: Hypoxia-inducible factor 1A
ICC: Intrahepatic cholangiocarcinoma
ICI: Immune checkpoint inhibitor
NAFLD: Non-alcoholic fatty liver disease
NAS: NAFLD activity score
NASH: Non-alcoholic steatohepatitis
OS: Overall survival
PET/CT: Positron emission tomography/computed tomography
PD-L1: Programmed death-ligand 1
RFS: Recurrence-free survival
ROC: Receiver operating characteristic
SUVmax: Maximum standardized uptake value
TILs: Tumor-infiltrating lymphocytes
TME: Tumor microenvironment

## References

[1] Siegel R, Ma J, Zou Z, Jemal A (2014). Cancer statistics, 2014. CA Cancer J Clin..

[2] Patel T (2001). Increasing incidence and mortality of primary intrahepatic cholangiocarcinoma in the United States. Hepatology.

[3] Banales JM, Cardinale V, Carpino G, Marzioni M, Andersen JB, Invernizzi P (2016). Expert consensus document: Cholangiocarcinoma: current knowledge and future perspectives consensus statement from the European Network for the Study of Cholangiocarcinoma (ENS-CCA). Nat. Rev. Gastroenterol. Hepatol..

[4] Yugawa K, Itoh S, Kurihara T, Yoshiya S, Mano Y, Takeishi K (2019). Skeletal muscle mass predicts the prognosis of patients with intrahepatic cholangiocarcinoma. Am. J. Surg..

[5] Blüher M (2019). Obesity: global epidemiology and pathogenesis. Nat. Rev. Endocrinol..

[6] Calle EE, Rodriguez C, Walker-Thurmond K, Thun MJ (2003). Overweight, obesity, and mortality from cancer in a prospectively studied cohort of U.S. adults. N. Engl. J. Med..

[7] Lauby-Secretan B, Scoccianti C, Loomis D, Grosse Y, Bianchini F, Straif K (2016). Body fatness and cancer-viewpoint of the IARC Working Group. N. Engl. J. Med..

[8] Reilly SM, Saltiel AR (2017). Adapting to obesity with adipose tissue inflammation. Nat. Rev. Endocrinol..

[9] Liu R, Nikolajczyk BS (2019). Tissue immune cells fuel obesity-associated inflammation in adipose tissue and beyond. Front Immunol..

[10] Moon CM, Bang S, Chung JB (2011). The role of (18)F-fluorodeoxyglucose positron emission tomography in the diagnosis, staging, and follow-up of cholangiocarcinoma. Surg. Oncol..

[11] Mano Y, Aishima S, Kubo Y, Tanaka Y, Motomura T, Toshima T (2014). Correlation between biological marker expression and fluorine-18 fluorodeoxyglucose uptake in hepatocellular carcinoma. Am. J. Clin. Pathol..

[12] Shirabe K, Toshima T, Kimura K, Yamashita Y, Ikeda T, Ikegami T (2014). New scoring system for prediction of microvascular invasion in patients with hepatocellular carcinoma. Liver Int..

[13] Theze B, Bernards N, Beynel A, Bouet S, Kuhnast B, Buvat I (2015). Monitoring therapeutic efficacy of sunitinib using [(18)F]FDG and [(18)F]FMISO PET in an immunocompetent model of luminal B (HER2-positive)-type mammary carcinoma. BMC Cancer.

[14] Xu G, Sun L, Li Y, Xie F, Zhou X, Yang H (2019). The Clinicopathological and prognostic value of PD-L1 expression in cholangiocarcinoma: a meta-analysis. Front Oncol..

[15] Yugawa, K., Itoh, S., Yoshizumi, T., Iseda, N., Tomiyama, T., Toshima, T., et al. Prognostic impact of tumor microvessels in intrahepatic cholangiocarcinoma: association with tumor-infiltrating lymphocytes. *Mod. Pathol.* (2020). 10.1038/s41379-020-00702-9 33077921

[16] Asahi, Y., Hatanaka, K. C., Hatanaka, Y., Kamiyama, T., Orimo, T., Shimada, S., et al. Prognostic impact of CD8+ T cell distribution and its association with the HLA class I expression in intrahepatic cholangiocarcinoma. *Surg Today*. (2020). 10.1007/s00595-020-01967-y 32040618

[17] Jing CY, Fu YP, Yi Y, Zhang MX, Zheng SS, Huang JL (2019). HHLA2 in intrahepatic cholangiocarcinoma: an immune checkpoint with prognostic significance and wider expression compared with PD-L1. J. Immunother. Cancer.

[18] Kuriyama K, Higuchi T, Yokobori T, Saito H, Yoshida T, Hara K (2020). Uptake of positron emission tomography tracers reflects the tumor immune status in esophageal squamous cell carcinoma. Cancer Sci..

[19] Hirakata T, Fujii T, Kurozumi S, Katayama A, Honda C, Yanai K (2020). FDG uptake reflects breast cancer immunological features: the PD-L1 expression and degree of TILs in primary breast cancer. Breast Cancer Res. Treat..

[20] Mitchell, K. G., Amini, B., Wang, Y., Carter, B. W., Godoy, M., Parra, E. R., et al. 18F-fluorodeoxyglucose positron emission tomography correlates with tumor immunometabolic phenotypes in resected lung cancer [published online ahead of print, 2020 Apr 16]. *Cancer Immunol. Immunother*. (2020). 10.1007/s00262-020-02560-5 PMC7997043 32300858

[21] Kleiner DE, Brunt EM, Van Natta M, Behling C, Contos MJ, Cummings OW (2005). Design and validation of a histological scoring system for nonalcoholic fatty liver disease. Hepatology.

[22] Sanyal AJ, Brunt EM, Kleiner DE, Kowdley KV, Chalasani N, Lavine JE (2011). Endpoints and clinical trial design for nonalcoholic steatohepatitis. Hepatology.

[23] Ogawa W, Miyazaki S (2015). Diagnosis criteria for obesity and obesity disease. Health Eval. Promot..

[24] Japan Society for the Study of Obesity. Guidelines for the Management of Obesity Disease 2016. Tokyo, Japan. Life Science Publishing.

[25] Itoh S, Yoshizumi T, Kimura K, Okabe H, Harimoto N, Ikegami T (2016). Effect of sarcopenic obesity on outcomes of living-donor liver transplantation for hepatocellular carcinoma. Anticancer Res..

[26] Itoh S, Yoshizumi T, Mori M (2020). Is sarcopenic obesity superior to sarcopenia as a predicting indicator in patients with hepatocellular carcinoma following hepatic resection?. Hepatobiliary Surg. Nutr..

[27] Shinkawa H, Tanaka S, Takemura S, Ito T, Aota T, Koda M (2018). Obesity and recurrence-free survival in patients with hepatocellular carcinoma after achieving sustained virological response to interferon therapy for chronic hepatitis C. Ann. Gastroenterol. Surg..

[28] Merath K, Mehta R, Hyer JM, Bagante F, Sahara K, Alexandrescu S (2019). Impact of body mass index on tumor recurrence among patients undergoing curative-intent resection of intrahepatic cholangiocarcinoma- a multi-institutional international analysis. Eur. J. Surg. Oncol..

[29] Sheka AC, Adeyi O, Thompson J, Hameed B, Crawford PA, Ikramuddin S (2020). Nonalcoholic steatohepatitis: a review. JAMA.

[30] Wongjarupong N, Assavapongpaiboon B, Susantitaphong P, Cheungpasitporn W, Treeprasertsuk S, Rerknimitr R (2017). Non-alcoholic fatty liver disease as a risk factor for cholangiocarcinoma: a systematic review and meta-analysis. BMC Gastroenterol..

[31] Fu K, Yang X, Wu H, Gong J, Li X (2020). Diabetes and PKM2 affect prognosis in patients with intrahepatic cholangiocarcinoma. Oncol. Lett..

[32] Dick KJ, Nelson CP, Tsaprouni L, Sandling JK, Aïssi D, Wahl S (2014). DNA methylation and body-mass index: a genome-wide analysis. Lancet.

[33] Wahl S, Drong A, Lehne B, Loh M, Scott WR, Kunze S (2017). Epigenome-wide association study of body mass index, and the adverse outcomes of adiposity. Nature.

[34] O'Sullivan J, Lysaght J, Donohoe CL, Reynolds JV (2018). Obesity and gastrointestinal cancer: the interrelationship of adipose and tumour microenvironments. Nat. Rev. Gastroenterol. Hepatol..

[35] Togo M, Yokobori T, Shimizu K, Handa T, Kaira K, Sano T (2020). Diagnostic value of (18)F-FDG-PET to predict the tumour immune status defined by tumoural PD-L1 and CD8(+)tumour-infiltrating lymphocytes in oral squamous cell carcinoma. Br. J. Cancer.

[36] Vansteenkiste JF, Stroobants SG, Dupont PJ, De Leyn PR, Verbeken EK, Deneffe GJ (1999). Prognostic importance of the standardized uptake value on (18)F-fluoro-2-deoxy-glucose-positron emission tomography scan in non-small-cell lung cancer: an analysis of 125 cases. Leuven Lung Cancer Group. J. Clin. Oncol..

[37] Brahmer JR, Tykodi SS, Chow LQ, Hwu WJ, Topalian SL, Hwu P (2012). Safety and activity of anti-PD-L1 antibody in patients with advanced cancer. N. Engl. J. Med..

[38] Gabrielson A, Wu Y, Wang H, Jiang J, Kallakury B, Gatalica Z (2016). Intratumoral CD3 and CD8 T-cell densities associated with relapse-free survival in HCC. Cancer Immunol. Res..

[39] Itoh S, Yugawa K, Shimokawa M, Yoshiya S, Mano Y, Takeishi K (2019). Prognostic significance of inflammatory biomarkers in hepatocellular carcinoma following hepatic resection. BJS Open.

[40] Itoh, S., Yoshizumi, T., Yugawa, K., Imai, D., Yoshiya, S., Takeishi, K., et al. Impact of Immune response on outcomes in hepatocellular carcinoma: association with vascular formation. *Hepatology* **72**(6), 1987–1999 (2020). 10.1002/hep.31206 32112577

[41] Wu X, Huang Y, Zhao Q, Wang L, Song X, Li Y (2020). PD-L1 expression correlation with metabolic parameters of FDG PET/CT and clinicopathological characteristics in non-small cell lung cancer. EJNMMI Res..

[42] Toyokawa G, Takada K, Okamoto T, Kozuma Y, Matsubara T, Haratake N (2017). Elevated metabolic activity on (18)F-FDG PET/CT is associated with the expression of EZH2 in non-small cell lung cancer. Anticancer Res..

[43] Yugawa K, Itoh S, Yoshizumi T, Yoshiya S, Takeishi K, Toshima T (2020). Prognostic impact of 8-hydroxy-deoxyguanosine and its repair enzyme 8-hydroxy-deoxyguanosine DNA glycosylase in hepatocellular carcinoma. Pathol. Int..

