# Supplementary material for: Obesity is a risk factor for intrahepatic cholangiocarcinoma progression associated with alterations of metabolic activity and immune status
Source: Sci Rep. 2021 Mar 12;11:5845. doi: 10.1038/s41598-021-85186-6 (PMC7955092; doi:10.1038/s41598-021-85186-6)
Supplement: Supplementary file 1 — Supplementary Information 1. [file 41598_2021_85186_MOESM1_ESM.pdf]

Obesity is a risk factor for intrahepatic cholangiocarcinoma progression associated with alterations of metabolic activity and immune status

Kyohei Yugawa, MD, PhD[1,#], Shinji Itoh, MD, PhD[1,#,*], Norifumi Iseda, MD[1], Takeshi Kurihara, MD, PhD[1], Yoshiyuki Kitamura, MD, PhD[3], Takeo Toshima, MD, PhD[1], Noboru Harada, MD, PhD [1], Kenichi Kohashi, MD, PhD[2], Shingo Baba, MD, PhD[3], Kousei Ishigami, MD, PhD[3], Yoshinao Oda, MD, PhD[2], Tomoharu Yoshizumi, MD, PhD[1], and Masaki Mori, MD, PhD[1]

[1]Department of Surgery and Science, Graduate School of Medical Sciences, Kyushu University, Fukuoka 812-8582, Japan

[2]Department of Anatomic Pathology, Graduate School of Medical Sciences, Kyushu University, Fukuoka 812-8582, Japan

[3]Department of Clinical Radiology, Graduate School of Medical Sciences, Kyushu University, Fukuoka 812-8582, Japan

[#]These authors contributed equally

***Corresponding author:** Shinji Itoh, MD, PhD, Department of Surgery and Science, Graduate School of Medical Sciences, Kyushu University, Fukuoka 812-8582, Japan

Tel: +81-92-642-5466

Fax: +81-92-642-5482

E-mail: itoshin@surg2.med.kyushu-u.ac.jp

Supplementary Figure 1

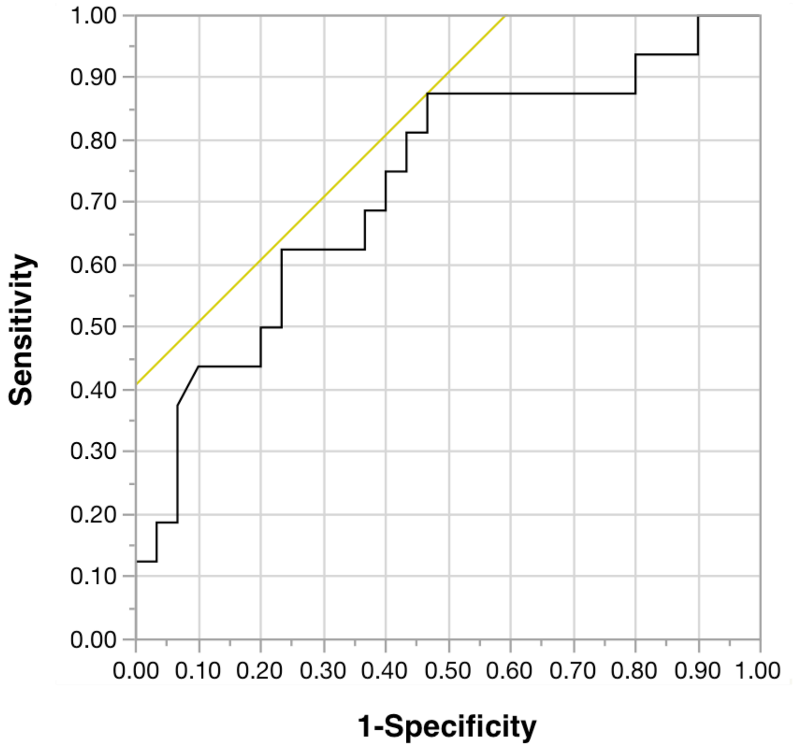

Supplementary Figure 1. Receiver operating characteristic (ROC) curve analysis for the optimal cutoff of the maximum standardized uptake value for fluorine-18 fluorodeoxyglucose on positron emission tomography/computed tomography. The area under the ROC curve was 0.728. The sensitivity and specificity were 87.5 and 53.3%, respectively.

Supplementary Figure 2

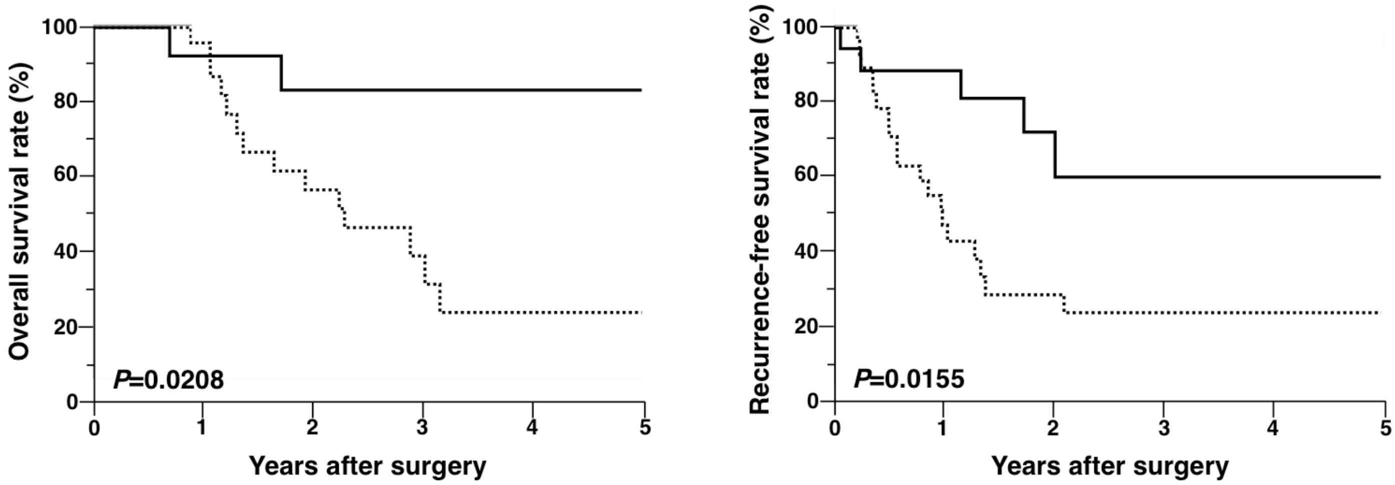

Number at risk

Years	0	1	2	3	4	5
SUVmax<6.79	18	13	8	5	5	4
SUVmax≥6.79	28	24	12	6	4	2

Number at risk

Years	0	1	2	3	4	5
SUVmax<6.79	18	13	7	5	5	3
SUVmax≥6.79	28	13	7	5	5	2

Supplementary Figure 2. Overall and recurrence-free survival rates after hepatic resection for intrahepatic cholangiocarcinoma in patients with high and low maximum standardized uptake values (SUVmax).

Supplementary Figure 3

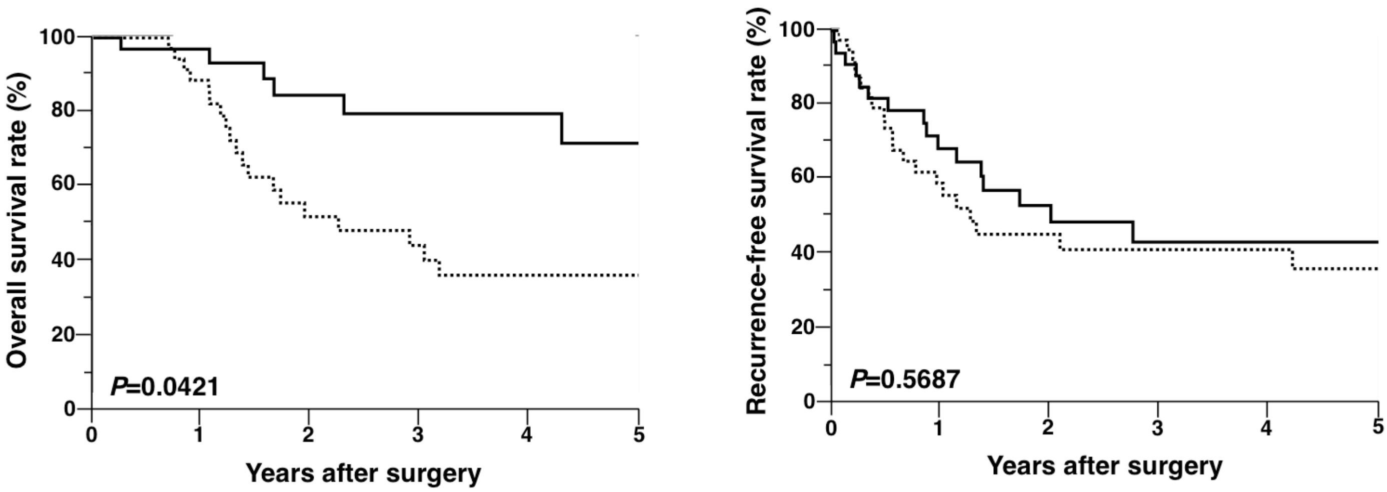

Number at risk

Years	0	1	2	3	4	5
PD-L1 negative	33	27	19	13	11	9
PD-L1 positive	41	32	15	12	10	7

Number at risk

Years	0	1	2	3	4	5
PD-L1 negative	33	21	13	9	8	6
PD-L1 positive	41	21	12	10	10	7

Supplementary Figure 3. Overall and recurrence-free survival rates after hepatic resection for intrahepatic cholangiocarcinoma in patients with programmed death-ligand 1 (PD-L1) + and PD-L1– tumors.
